# Influence of platelet count at diagnosis and during the course of disease on prognosis in MDS patients

**DOI:** 10.1007/s00277-021-04608-7

**Published:** 2021-07-29

**Authors:** Judith Strapatsas, Elena Calina Barbulescu, Michael Lauseker, Jennifer Kaivers, Barbara Hildebrandt, Kathrin Nachtkamp, Corinna Strupp, Martina Rudelius, Rainer Haas, Ulrich Germing

**Affiliations:** 1 Department of Hematology, Oncology and Clinical Immunology, Heinrich-Heine University, Moorenstr. 5, 40225 Düsseldorf, Germany; 2 Institute for Medical Information Sciences, Biometry and Epidemiology, Ludwig-Maximilians-University, Munich, Germany; 3 Department of Human Genetics, Heinrich-Heine University, Duesseldorf, Germany; 4 Department of Oncology, Rheinland Klinikum, Dormagen, Germany; 5 Institute for Pathology, Ludwig-Maximilians-University, Munich, Germany

**Keywords:** Thrombocytopenia, Prognosis, Platelet drop, Morphology, Bleeding, MDS

## Abstract

Thrombocytopenia at diagnosis and platelet drop within the first 6 months have an adverse effect on prognosis of MDS patients. We therefore were interested in the association and impact on prognosis of morphologic findings of megakaryocytes and platelets with platelet count at diagnosis, bleeding complications, and the drop of platelets during the course of disease. This retrospective analysis was based on 334 MDS patients from the Duesseldorf MDS registry that were followed up for blood counts, bleeding, transfusion dependency, and AML evolution and correlated with morphology of the megakaryocytes and platelets. Thrombocytopenia was found more frequently in higher risk MDS and was associated with hypocellularity of the megakaryocytes in the bone marrow. Signs of bleeding were present at diagnosis in 14% and occurred during the disease in 48% of all MDS patients. Death due to bleeding was ranked third behind infections and AML. A decrement of platelets during the first 6 months was associated with an inferior overall survival of 21 vs. 49 months and with a higher cumulative 2-year AML rate of 22.2% vs. 8.3% (*p* = 0.001). In a multivariate analysis, besides bone marrow blasts and karyotype, decreasing platelets were also associated with an inferior outcome. Signs of bleeding are present in a relevant number of MDS patients and account for significant morbidity and mortality in MDS. We could demonstrate the prognostic importance of decreasing platelets during the course of disease in all MDS patients, identifying patients at higher risk for death or AML progression.

## Introduction

Myelodysplastic syndromes are heterogeneous stem cell disorders characterized by dysplastic features in the bone marrow and various degrees of cytopenia in the peripheral blood that have been identified as an important prognostic parameter. For prognostication of patients with MDS, the International Prognostic Scoring System (IPSS) is used as the gold standard and was revised in 2012 [1, 2].

About 35–67% of all MDS patients present with thrombocytopenia at the time of diagnosis, whereas isolated thrombocytopenia occurs in less than 10% [3–6]. Thrombocytopenia in MDS is mainly caused by ineffective platelet production as a result of disturbed proliferation and maturation of megakaryocytes or their precursors [7]. Cytogenetic studies revealed that even in the case of a normal appearance on light microscopy, the majority of megakaryocytes are part of the MDS clone [8]. Although it is well known that the initial platelet count is an important prognostic parameter, data on the clinical course of the patients with low platelets during the course of the disease are sparse. Besides AML evolution, infections and bleeding complications are the most frequent causes of death [9]. Death due to bleeding complications is found not only in patients with low platelet counts but also in patients with higher or even normal platelets, suggesting underlying platelet dysfunction [5, 10]. There is evidence that the kinetics of cytopenia in lower risk MDS patients and the occurrence of red blood cell transfusion dependency have a prognostic impact [11, 12]. To better elucidate the influence of transfusion dependency at the time of diagnosis, during the course of the disease and, most importantly, the prognostic impact with regard to survival and risk of progression to AML of decreasing platelet counts during the course of the disease across all MDS subtypes are the major aims of this study. We correlated these findings with morphological findings in the blood and in the bone marrow.

## Methods

This retrospective analysis is based on the Düsseldorf MDS registry, which was initiated in 1982 and maintained since then. Diagnostic procedures were the same as reported in our previous studies [13, 14]. In total, 334 of patients included in the registry between 2008 and 2015 were followed up closely for blood counts every 3 months including morphology of the platelets in the differential blood count, transfusion need, and for survival and AML evolution. Only patients with at least one follow-up blood count were included. All blood and bone marrow smears were examined by the same investigator (U.G.) at our hematologic laboratory. The morphological diagnosis was made according to the proposals of the 2016 WHO classification [15]. Patients with CMML were also included in the analysis. A peripheral blood smear was examined to determine the differential white blood count, recognize circulating blast cells, and assess platelet morphology. In the marrow, a differential count of 500 nucleated cells was performed to determine the proportion of medullary blasts. Megakaryopoiesis was analyzed with regard to maturation defects and dysplastic features of megakaryocytes. Dysmegakaryopoiesis was diagnosed if at least 10 out of 25 megakaryocytes were micromegakaryocytes or mononuclear megakaryocytes, or had multiple widely separated nuclei. Maturation defects included all other features representing disturbed maturation without clear dysmegakaryopoietic criteria as previously described [5]. Cytogenetic analysis by metaphase karyotyping was performed in the Institute of Human Genetics, Heinrich-Heine University, Duesseldorf. An isolated thrombocytopenia was considered if the platelet count was < 100,000/µl, the hemoglobin value was > 10 g/dl, and the ANC was > 800/µl. If possible, patients were followed for platelet transfusion requirement triggered by bleeding signs or thrombocytopenia < 10,000/µl, signs of bleeding, and causes of death. Patients were considered platelet transfusion dependent if platelets had to be administered at least once per month. Transfusion requirement during allogeneic stem cell transplantation, treatment with hypomethylating agents, or induction chemotherapy was not regarded as transfusion dependency and therefore was not considered for the analysis. For survival analysis, patients who received allogeneic stem cell transplantation (*n* = 55, 16.5%) were censored at the time of transplantation. Survival probabilities were estimated using the Kaplan–Meier method and compared by the log-rank test, when no time-dependent covariates were present. To investigate the impact of a decrease of platelet counts, a landmark analysis was performed 6 months after diagnosis (± 1 months) [16]. This analysis included those patients, who survived for more than 7 months, who had a second blood count within this time and had an initial platelet count > 20,000/µl and were not transfusion dependent. Cumulative incidences of AML were estimated under the presence of the competing risk of death. For the multivariate models, missing covariate values were imputed by fully conditional specification: accommodating the substantive model. One hundred datasets were generated using 25 iterations each [17]. Clinical and hematological data at the time of diagnosis were compared using the *χ*-square and Wilcoxon rank-sum test. A two-sided *p*-value of less than 0.05 was considered statistically significant. For all statistical analyses, SPSS version 25 and R 3.5.1 were used.

## Results

### Patient characteristics

Looking at the entire cohort of 334 patients, 199 were male (60%) and the median age was 68 years (range 22–87). After a median follow-up of 24 months (range 1–187 months), 161 patients have died (48%). From the entire patient cohort, 76 (23%) patients progressed into acute myeloid leukemia (AML). When categorizing patients according to the initial platelet count using the IPSS-R cutoffs, 106 patients presented with platelets > 100,000/µl (32%), 122 had a platelet count between 50,000 and 100,000/µl (37%), and 101 patients (31%) presented with platelets < 50,000/µl, respectively. Only 26 patients (7.8%) had severe thrombocytopenia of < 20,000/µl. Baseline characteristics are presented in Table 1. An isolated thrombocytopenia at the time of diagnosis was present in 77 patients (21%).

**Table 1:** Baseline characteristics

	All *n* = 334	Thrombocytes/µl			TD at diagnosis *n* = 45
		> 100,000 *n* = 107	50–100,000 *n* = 125	< 50,000 *n* = 102	
Sex male	199 (60%)	59 (55%)	83 (66%)	57 (56%)	29 (64%)
Female	135 (40%)	48 (45%)	42 (34%)	45 (44%)	16 (36%)
med. age	68 (23–87)	68 (45–87)	69 (23–87)	65 (35–83)	66 (39–84)
WHO	*n* = 324				*n* = 45
SLD*	17 (5.1%)	6 (5.6%)	4 (3.3%)	7 (7.1%)	1 (2.2%)
MLD*	134 (41.4%)	49 (45.8%)	44 (37.0%)	41 (41.8%)	17 (37.8%)
5q-	11 (3.4%)	9 (8.4%)	2 (1.7%)	0	0
EB-1	52 (16.0%)	14 (13.1%)	28 (23.5%)	10 (10.2%)	5 (11.1%)
EB-2	62 (19.1%)	12 (11.2%)	23 (19.3%)	27 (27.6%)	13 (28.9%)
CMML-1	30 (9.3%)	14 (13.1%)	12 (10.1%)	4 (4.1%)	3 (6.7%)
CMML-2	12 (3.7%)	2 (1.9%)	5 (4.2%)	5 (5.1%)	4 (8.9%)
MDS-U	6 (1.8%)	1 (0.9%)	1 (0.8%)	4 (4.1%)	2 (4.4%)
IPSS-R	*n* = 256				*n* = 34
Very low	27 (10.5%)	16 (19%)	10 (10.2%)	1 (1.4%)	1 (2.9%)
Low	66 (25.8%)	35 (41.7%)	15 (15.3%)	16 (21.6%)	3 (8.8%)
Intermediate	60 (23.4%)	20 (23.8%)	28 (28.6%)	12 (16.2%)	6 (17.6%)
High	51 (19.9%)	10 (11.9%)	21 (21.4%)	20 (27.0%)	10 (29.4%)
Very high	52 (20.3%)	3 (3.6%)	24 (24.5%)	25 (33.8%)	14 (41.2%)

*TD* transfusion dependency

^*^Patients with SLD-RS (*n* = 6) and MLD-RS (*n* = 2) are included in SLD and MLD

Looking at the distribution of patients according to the WHO 2016 classification, we found in the unfavorable risk category MDS EB-2 a high number of patients with decreasing initial platelet counts. In accordance to this finding, the amount of higher risk MDS according to the IPSS-R also increased with decreasing initial platelet count. The correlation of increasing thrombocytopenia and increasing risk category according to the IPSS-R was even more evident when looking at the small group of patients with transfusion dependency at the time of diagnosis with 41% classified as very high risk.

Forty-five patients (14%) were transfusion dependent at the time of diagnosis and 92 patients (27%) developed transfusion dependency during the course of the disease. Information about the dimension of transfusion dependency was available in 297 patients. The median number of transfused platelet units was 17 (range 1–177). A total of 133 patients received none and 133 patients 1–50 platelet units; 31 patients were heavily transfused with > 50 units.

A platelet decrement of at least 25% from the initial platelet count within the first 6 months after diagnosis (± 1 months) was found in 70 patients (21%). In this cohort, we also found a higher number of patients with higher risk MDS than in those patients without decreasing platelets (Table 2).

**Table 2:** Characteristics of patients included into the landmark analysis

	All *n* = 146	No platelet decrement *n* = 76	Platelets decreased 25% *n* = 70
Sex male	89 (61%)	45 (59%)	44 (63%)
Female	57 (39%)	31 (41%)	26 (37%)
med. age	69 (42–84)	68 (45–84)	69 (42–84)
WHO	*n* = 143	*n* = 74	*n* = 69
SLD	6 (4.2%)	4 (5.4%)	2 (2.9%)
MLD	61 (42.7%)	33 (44.6%)	28 (40.6%)
5q-	6 (4.2%)	5 (6.8%)	1 (1.4%)
EB-1	22 (15.4%)	15 (20.3%)	7 (10.1%)
EB-2	25 (17.5%)	9 (12.2%)	16 (23.2%)
CMML-1	15 (10.5%)	5 (6.8%)	10 (14.5%)
CMML-2	6 (4.2%)	3 (4.1%)	3 (4.3%)
MDS-U	2 (1.4%)	0	2 (2.9%)
IPSS-R	*n* = 118	*n* = 58	*n* = 60
Very low	12 (10.2%)	7 (12.1%)	5 (8.3%)
Low	34 (28.8%)	21 (36.2%)	13 (21.7%)
Int	28 (23.7%)	13 (22.4%)	15 (25.0%)
High	24 (20.3%)	11 (19.0%)	13 (21.7%)
Very high	20 (17.0%)	6 (10.3%)	14 (23.3%)

### Bleeding

Information about signs of bleeding at the time of diagnosis and during follow-up were available from 172 (52%) and 193 (58%) patients, respectively. Among those, 24 patients (14%) showed signs of bleeding at diagnosis and 92 patients (48%) developed bleeding complications during the follow-up period. Among both groups, petechia was the most frequently observed sign of bleeding (54% and 36%, respectively). There was no correlation between IPSS-R risk category and the presence of bleeding at diagnosis nor during the disease. However, as expected, the initial platelet count was correlated with signs of bleeding at the time of diagnosis (*p* = 0.002). When looking at the patients with a drop of the platelet count and an occurrence of bleeding, 69 (61%) developed bleeding complications during the course of the disease.

Causes of death were known from 120 patients (75%). Death was disease related in 95%, with death due to infection as the most frequent cause of death in 35%, followed by death due to AML in 19%. Fatal bleeding complications occurred in 15 patients (9.3%). Of note, only one patient who presented with signs of bleeding at diagnosis died due to bleeding complications. The majority of patients that developed signs of bleeding during the course of the disease died due to other reasons (*n* = 48, 76%). Looking at those patients with isolated thrombocytopenia, causes of death were comparable to patients with a bi- or tricytopenia.

### Platelet count and morphology

In the next step, we investigated a potential correlation between platelet counts and morphological findings. The distribution of morphological findings in the peripheral blood as well as in the bone marrow according to the platelet count is shown in Table 3. Of note, we found a significant correlation between the histologic and cytologic analyses when looking at the cellularity of the megakaryopoiesis (OR 14.54; 95%CI 6.03–35.07). Additionally, hypocellular megakaryopoiesis was significantly correlated with thrombocytopenia (OR 4.29, 95%CI 2.55–7.21). Of note, we could not find a correlation between the cellularity of the megakaryopoiesis and the presence of dysmegakarypoiesis, but the presence of dysmegakaryopoiesis in the bone marrow reflects an abnormal platelet morphology in the peripheral blood (OR 2.95; 95%CI 1.03–8.44), although we could not identify a specific dysplastic feature of the megakaryocytes associated with abnormal platelet morphology. Morphologically abnormal platelets were correlated with transfusion dependency at diagnosis (OR 10.03; 95%CI 1.29–78.11). The presence of signs of bleeding at diagnosis as well as the frequency of AML progression correlated neither with dysplastic features of the megakaryopoiesis nor with platelet morphology or cellularity of the megakaryocytes at diagnosis.

**Table 3:** Morphology of thrombocytes in the peripheral blood and megakaryocytes

	All	Thrombocytes/µl		
		> 100,000	50–100,000	< 50,000
Suspicious platelet morphology	48 (14.4%)	28 (63.6%)	14 (26.9%)	6 (14.0%)
Platelet anisometry	39 (11.7%)	24 (22.4%)	11 (8.8%)	4 (3.9%)
Giant platelets	26 (7.8%)	13 (12.1%)	9 (7.2%)	4 (3.9%)
Any sign of dysmegakapoiesis	204 (61.1%)	66 (76.7%)	74 (71.8%)	64 (72.7%)
Maturation defects	81 (24.3%)	25 (29.1%)	24 (24.2%)	32 (37.6%)
Micromegakaryocytes	86 (25.7%)	28 (32.6%)	33 (32.7%)	25 (29.1%)
Mononuclear forms	95 (28.4%)	32 (37.2%)	35 (35%)	28 (32.9%)
Multiple separated nuclei	97 (29.0%)	30 (34.9%)	33 (33.3%)	34 (40.5%)
Megakaryocytic cellularity	n = 285	n = 89	n = 105	n = 93
Hypocellular	88 (30.9%)	14 (15.7%)	24 (22.9%)	50 (54.9%)
Normocellular	123 (43.2%)	47 (52.8%)	48 (45.7%)	28 (30.8%)
Hypercellular	74 (26.0%)	28 (31.5%)	33 (31.4%)	13 (14.3%)

Information on platelet morphology at diagnosis and at the time when platelets decreased was available from 47 patients. In this small cohort, we were not able to find a correlation between the morphology of the platelets and the progression of thrombocytopenia at the two times.

### Prognosis

**Fig. 1:**
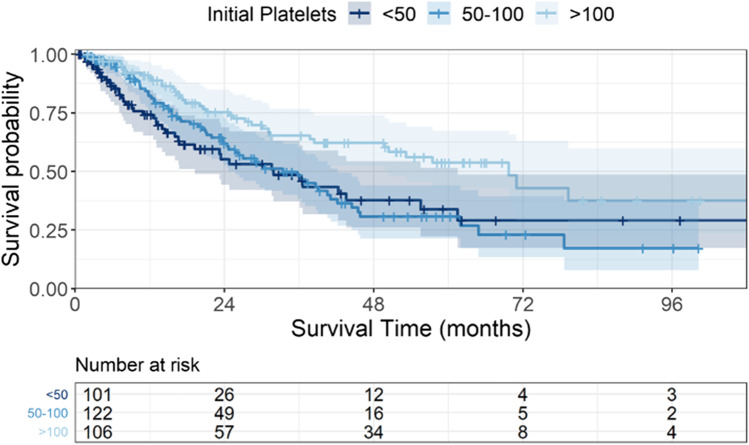
Overall survival according to initial platelet count. Cumulative survival probability according the to the initial platelet count. Patients with platelet counts between 50,000 and 100,000/µl, and with platelet counts < 50,000/µl had a significantly worse survival probability with medians of 33 and 31 months in comparison to a normal platelet count with 70 months OS (*p* = 0.003)

Patients with a normal platelet count had the best prognosis with a median survival of 70 months. In comparison, patients with platelet counts between 50,000 and 100,000/µl, and with platelet counts < 50,000/µl had a significantly worse survival probability with medians of 33 and 31 months (*p* = 0.003, Fig. 1). In line with this finding, platelet transfusion dependency at the time of diagnosis decreased survival probabilities in our cohort significantly (*p* = 0.02) with a median survival of 15 vs. 37 months, respectively. An isolated thrombocytopenia at the time of diagnosis was associated with a better prognosis in comparison to bi- or tricytopenia (median survival: 46 vs. 24 months, *p* < 0.0001). The presence of dysmegakaryopoietic features in the bone marrow was also correlated with prognosis (29 vs. 65 months, *p* = 0.02), whereas megakaryocytic cellularity, dysmorphologic features of the thrombocytes, and the presence of bleeding at diagnosis showed a tendency for inferior survival without being statistically significant (data not shown).

After a median follow-up of 24 months, 76 of the patients had progressed into an AML. Median time to AML evolution was not reached, but after 2 years, the cumulative incidence was 23% (95%CI 17.9–27.8%).

### Prognostic impact of decreasing platelets

**Fig. 2:**
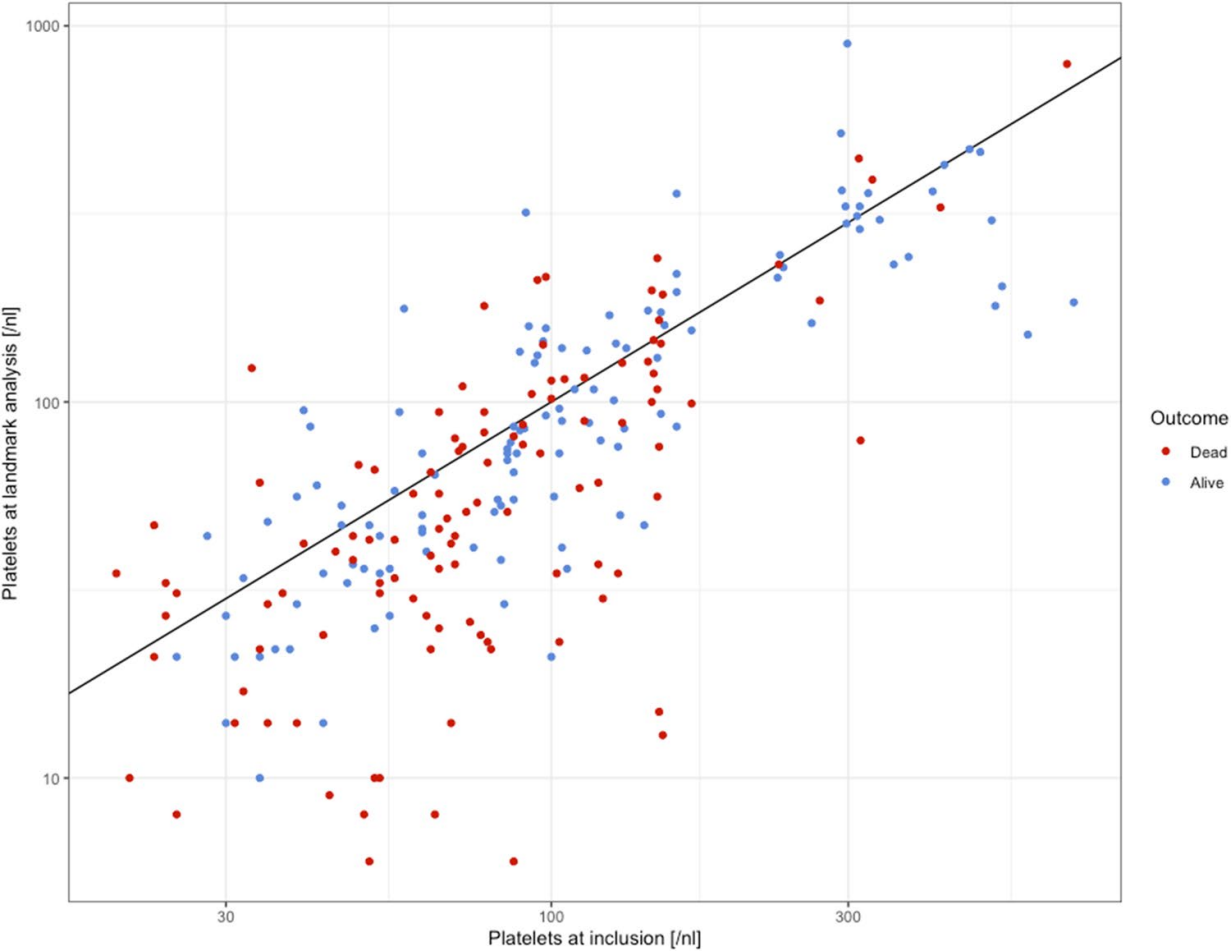
Initial platelet count and platelets at landmark depending on outcome. Scatter plot of the initial platelet count and platelets at landmark for each patient depending on outcome (alive in blue and dead in red)

**Fig. 3:**
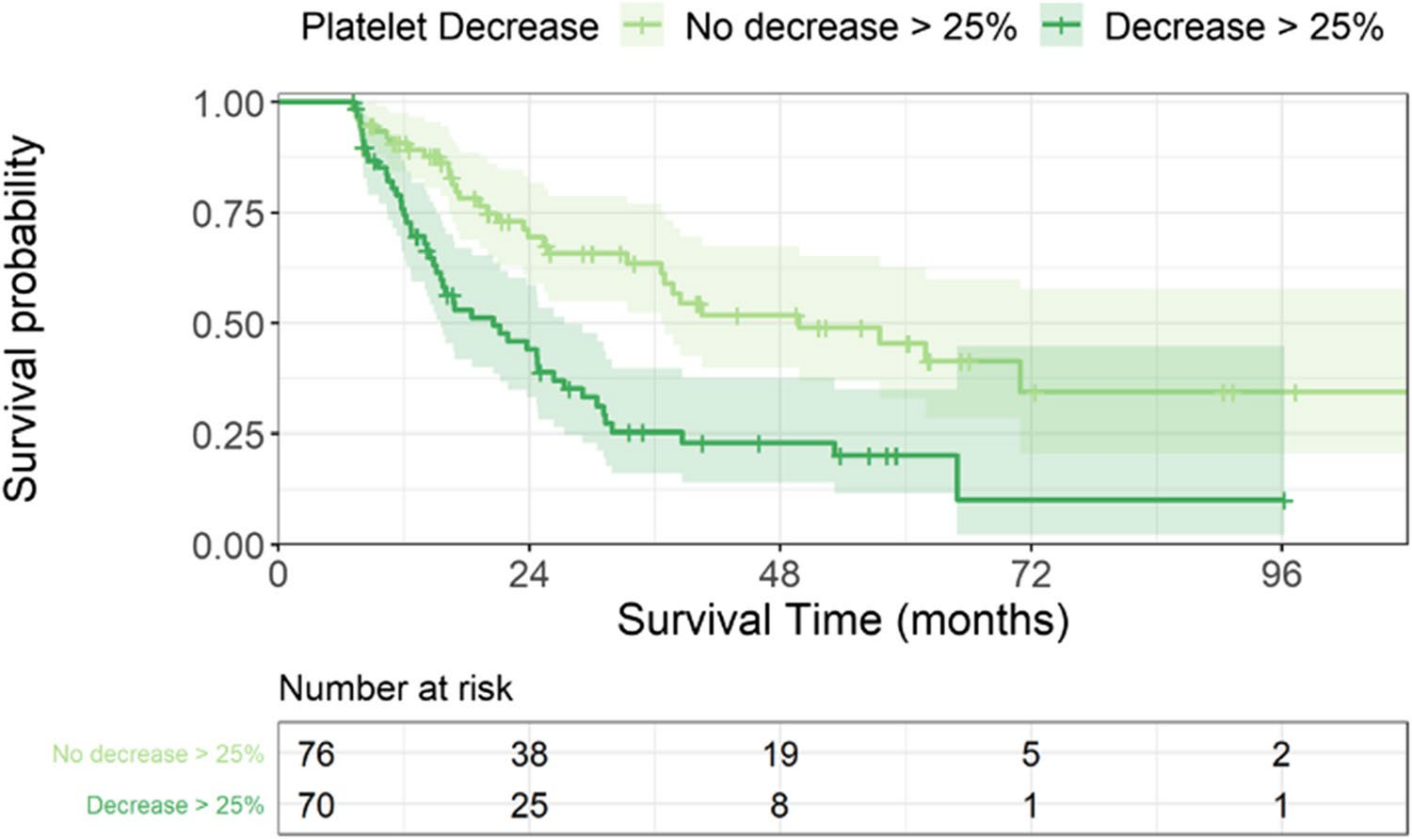
Overall survival after landmark of 6 months. Cumulative survival of patients with and without a platelet decrement. A platelet decrement of at least 25% within the first 6 months (± 1 months) after diagnosis was significantly associated with an inferior survival of 21 vs. 49 months, respectively (*p* < 0.001)

After demonstrating the prognostic relevance of the platelet count at diagnosis, we were interested in the prognostic impact of the decreasing platelets during the course of the disease. Performing a landmark analysis, we could demonstrate an inferior survival in patients with a platelet drop. We compared them with those patients who survived for more than 7 months, and who had a second blood count within this time and had an initial platelet count > 20,000/µl but remained stable with the platelet count. Figure 2 shows the initial platelet count and platelet count at landmark for all patients depending on outcome. This survival disadvantage was found in the entire patient cohort as well as when excluding patients receiving an allogeneic stem cell transplantation or induction chemotherapy (median survival: 49 vs. 21 months and 38 vs. 17 months, respectively, *p* < 0.001; Fig. 3). We then performed the landmark analysis separately for patients with lower risk MDS with a medullary blast count < 5% and for those with higher risk disease with a blast count > 5%. Median survival times for patients with < 5% BM blasts with platelet drop were 25 months and 58 months without platelet drop, respectively (*p* = 0.009). In the higher risk cohort, we could confirm the prognostic influence of an early platelet drop with median survival times of 15 and 33 months, respectively (*p* = 0.003).

**Fig. 4:**
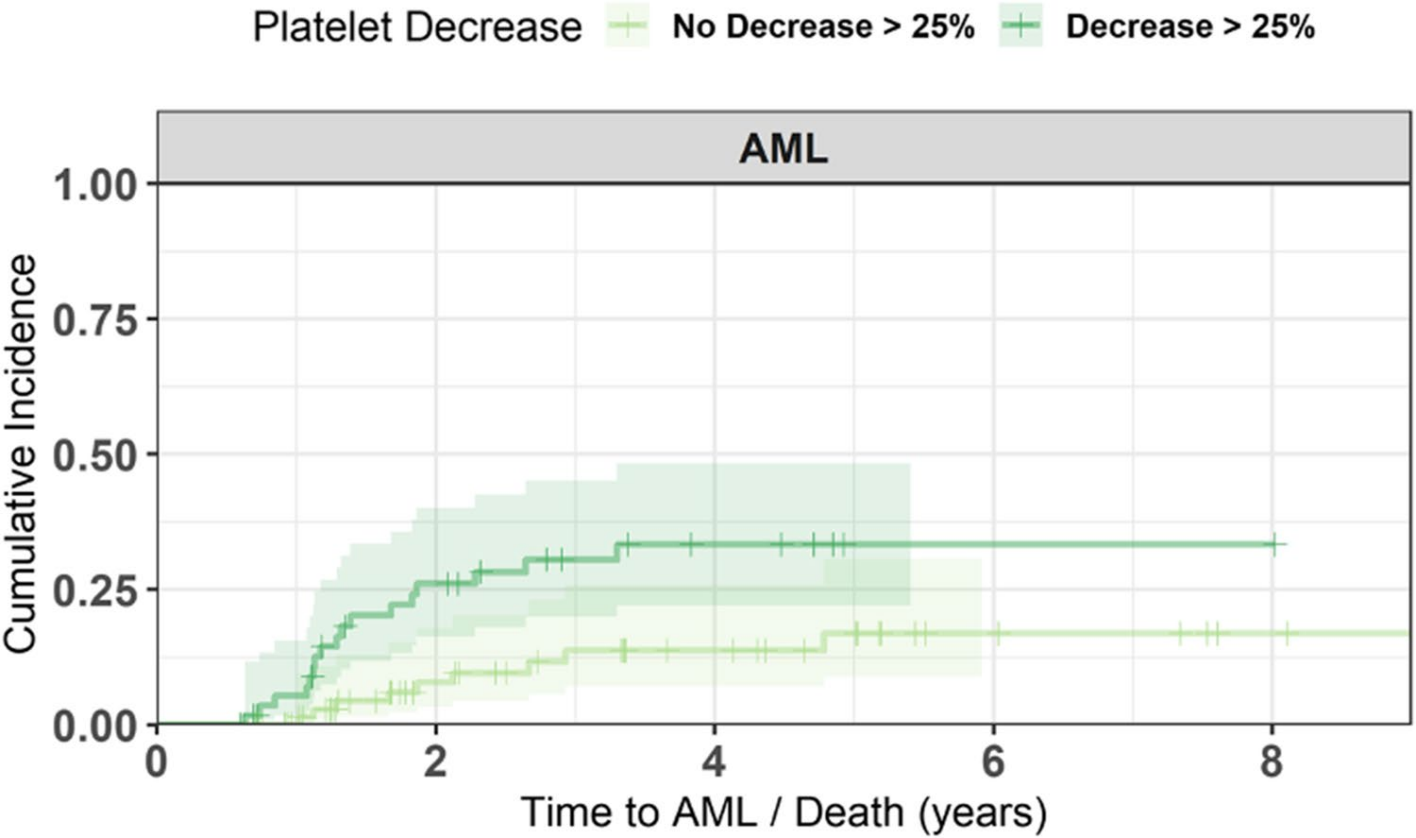
AML progression after landmark for all patients. Cumulative incidence of AML evolution in patients with and without platelet decrement. The 2-year cumulative incidence of AML evolution differed significantly between patients with decreasing platelets of at least 25% at landmark of 6 months and without decreasing platelets (8.3% vs. 22.2%, *p* < 0.001)

Within the first 6 months after diagnosis, 29 patients have progressed into AML. As we were interested in the clinical course of the disease in patients after our landmark, we excluded these patients from the following analysis. When only looking at those patients that developed AML later, the 2-year cumulative incidence of AML evolution differed significantly between those patients with decreasing platelets of at least 25% at landmark of 6 months and without decreasing platelets (22% [95%CI 14–35%] and 8.3% [95%CI 3.5–19%], *p* < 0.001, Fig. 4). When looking at the lower risk MDS patients, the 2-year cumulative incidence of AML evolution was 16% [95%CI 6.4–38%] for patients with decreasing platelets and 3.4% [95%CI 0.5–22%] for patients without decreasing platelets (*p* = 0.19). In patients with > 5% bone marrow blasts, AML incidences were 41% [95%CI 24–64%] vs. 18% [95%CI 7.1–41%], respectively (*p* = 0.09). AML progression was observed in 14 patients (21%) at the same time as the decrease of platelets, in 15 patients before the platelet decreased, and in 38 patients after the platelet decreased.

**Fig. 5:**
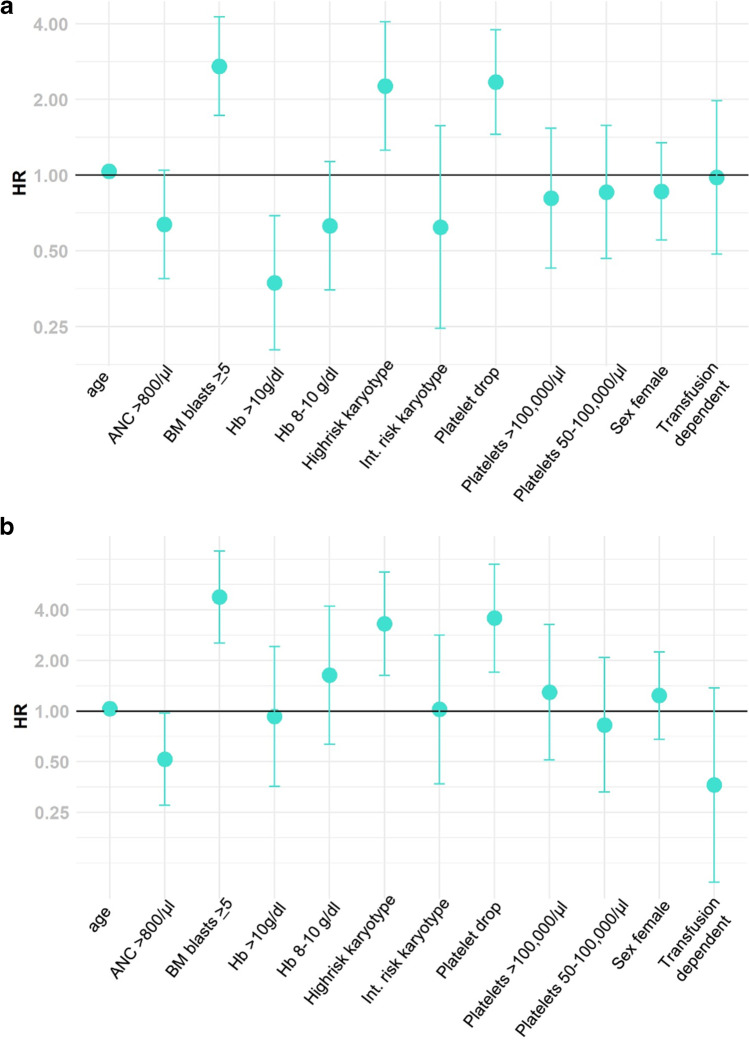
Forest plot of HR for overall survival (**a**) and AML evolution (**b**). **a** Significant predictors for survival were age > 65 years, a medullary blast count > 5%, a high-risk karyotype, and platelet drop > 25% within 6 months after diagnosis. **b** In a cause-specific hazard risk model for AML progression, platelet drop, a medullary blast count > 5%, and a high-risk karyotype were significantly correlated with AML evolution

To further elucidate the influence of decreasing platelets, we performed a multivariate analysis. We identified higher age (HR 1.04; 95%CI 1.01–1.06; *p* = 0.005), a medullary blast count > 5% (HR 2.71; 95%CI 1.73–4.26, *p* < 0.0001), and a higher risk karyotype (HR 2.26; 95%CI 1.26–4.07; *p* = 0.007) significantly predicting survival besides the platelet drop (HR 2.35; 95%CI 1.45–3.79, *p* = 0.0005). In a cause-specific hazard risk model for AML progression, platelet drop, a medullary blast count > 5%, and a high-risk karyotype were significantly correlated with AML evolution (HR 3.57; 95%CI 1.70–7.47, *p* = 0.0007; HR 4.76; 95%CI 2.53–8.96, *p* < 0.0001; and HR 3.30; 95%CI 1.63–6.69, *p* = 0.0009, respectively; Fig. 5).

## Discussion

Thrombocytopenia is a frequent finding in MDS patients. Therefore, with our study, we wanted to shed more light on the relationship between platelet counts, morphology, bleeding complications, and prognostic impact of platelet counts during the course of the disease. We could demonstrate a correlation between platelet count and cellularity of the megakaryocytes in the bone marrow. Furthermore, bleeding complications as well as fatal bleedings can occur in all MDS patients, not only in those with a severe thrombocytopenia. Third, an early drop of the platelet count during the course of the disease is a strong parameter for an adverse prognosis. To our knowledge, we are the first who could demonstrate the negative impact of platelet drop not only in low-risk patients as the Duesseldorf MDS registry allows a closer look on the platelet counts over the time of several different MDS patients.

This analysis is based on 334 MDS patients with 60% male patients, comparable to other studies [2, 11]. In our 334 MDS patients with available platelet counts and transfusion need during the disease, we could show that thrombocytopenia is a frequent finding in MDS patients with about 2/3 of the patients presenting with platelets of < 100,000 at the time of diagnosis. In our cohort, we found a higher number of patients with a thrombocytopenia than in other publications from Greenberg with 40% and Neukirchen with 43% [2, 5]. This finding may be explained by the fact that we focused on those patients who were seen regularly at our center and whose repeated blood counts were available. This closely followed patient cohort comprising a higher number of high-risk patients (50%). Likewise, the percentage of patients with an isolated thrombocytopenia (26%) was higher than that in previously published studies with 12% [6, 18]. Of note, in different studies published, varying cutoffs were used, especially, different hemoglobin levels of 10 g/dl and 12 g/dl, respectively. In our cohort, there was only a small number of patients with very severe thrombocytopenia < 20,000/µl. Therefore, an evaluation of those patients separately did not reveal additional information.

In MDS, there is no clear correlation between distinct dysplastic features in the bone marrow and peripheral blood counts, although with an increasing number of different signs of dysplasia the cell counts were lower [19]. We therefore analyzed the morphological features of our cohort in detail.

Although histology remains the gold standard for the evaluation of bone marrow, cellularity results of cytology and histology were comparable in our cohort, when restricting this analysis to the cellularity of the megakaryocytes. This fact may be due to an easy recognition of megakaryocytes in the bone marrow. The impact of bone marrow cellularity on prognosis is still a matter of debate. Some studies could show a different prognosis for patients with hypocellular bone marrow and suggest that hypocellular MDS could be considered a separate entity, whereas others did not [20–22]. Patients with a hypocellular bone marrow present more often with lower peripheral blood counts [22, 23]. When focusing on the cellularity of the megakaryocytes, we could demonstrate a clear correlation between hypocellularity of the megakaryocytes and thrombocytopenia in the peripheral blood as a result of the disturbed megakaryocytic differentiation and maturation. This finding proceeds with the occurrence of morphologically abnormal platelets in the peripheral blood. In summary, we can state that the evaluation of dysplastic features has an important impact on diagnostic procedures of MDS patients but is not an appropriate feature for the evaluation of the prognosis during the course of the disease. In contrast to our previous publication on a larger cohort, we could not demonstrate a correlation between signs of dysmegakaryopoiesis or dysplastic platelet morphology and signs of bleeding [5]. We suppose this is due to the relatively small cohort currently analyzed.

A platelet transfusion dependency at the time of diagnosis was found in 14% and occurred during the course of the disease in 27% of the patients, comparable to previously published data from the Duesseldorf MDS registry [5]. Although the aim of regular transfusion is the prevention of bleeding complications, 32% of all patients succumbed from signs of bleeding either at diagnosis or during the course of the disease and 13% died due to bleeding complications. The observed rate of death due to bleeding complications is also in line with our previously published data. As expected, platelet count at diagnosis, as well as thrombocytopenia during the course of the disease, was significantly correlated with the occurrence of bleeding. But, of note, even about 30% of the patients with higher platelet counts at diagnosis presented signs of bleeding during the disease. This finding reflects an additionally underlying platelet dysfunction in MDS patients that may also influence the risk of bleeding complications. The most common functional abnormality of the platelet function in MDS is impaired or absent aggregation; in detail, platelets of MDS patients show a general defect in integrin-dependent aggregation [10]. Remarkably, the majority of patients that developed signs of bleeding during the disease did not die due to fatal bleeding, maybe reflecting an effective transfusion regime. Nachtkamp et al. analyzed the causes of death in 2877 MDS patients. Death due to bleeding complications was with 9.8%, ranking third following AML and infections, too [9].

The prognostic relevance of thrombocytopenia in MDS patients is well known and could be confirmed by our data. Additionally, in line with previously published studies, we could show that overall survival is superior with the occurrence of isolated thrombocytopenia. This reflects a more aggressive disease, when more cell lines are affected. Besides known prognostic parameters as cell counts at diagnosis, we could show that the drop of platelets during the disease is an important factor associated with inferior prognosis regarding survival as well as AML transformation rates. Currently, only few data on the impact of worsening of the platelet count during the disease are available. On a large cohort from the European Leukemia Net MDS registry, a relative platelet drop > 25% and the presence of a transfusion dependency for red blood cells 6 months after diagnosis could identify lower risk MDS patients with an adverse prognosis [11]. In this work, a platelet drop within 6 months occurred in 21.7% of the low-risk patients. In our cohort, we observed a platelet drop in 48%, reflecting our cohort with low- and high-risk patients. We were able to confirm the prognostic relevance of a relative drop of platelets of ≥ 25% during the first 6 months not only in lower risk patients but also in higher risk patients. For the cumulative overall survival, an early platelet drop was significantly associated with an adverse prognosis in lower risk as well as in higher risk patients. Due to the smaller number of patients in our cohort than in the work from the EUMDS registry, we could not stratify for all IPSS-R subgroups. We therefore chose the bone marrow blast count of 5% as a cutoff for defining high- and low-risk disease. For AML progression, cumulative incidences at 2 years were lower in patients without a platelet drop irrespective of the bone marrow blast count. We obtained similar results, when setting the cutoff for the drop of platelets to 50% (data not shown). This negative impact on prognosis was demonstrated in untreated BSC patients as well as in the entire patient cohort that comprises patients treated with induction chemotherapy and hypomethylating agents, as well as allogeneic stem cell transplantation. Compared to the data from Itzykson and colleagues, the overall survival rates as well as the incidences of AML progression were higher in our cohort, reflecting the higher number of patients with high-risk MDS [11]. Factors associated with an early platelet drop in a multivariate analysis were higher age, higher risk IPSS-R, and initially existing thrombocytopenia. The drop of platelets within the first 6 months increased the cause-specific hazard for AML progression as well as the overall mortality hazard, reflecting the importance of changing parameters during the course of the disease. Of interest, when looking at the patients either with development of transfusion dependency for platelets or with an early drop of platelets and the IPSS-R categories at the time of diagnosis, we found no correlation between the IPSS-R risk and the worsening of the peripheral blood counts (data not shown). This finding can be regarded as a limitation of static risk models as the IPSS-R that cannot be adapted to the clinical course of each individual patient. Therefore, dynamic risk models that recognize disease parameters during the course of the disease of each patient are warranted to better identify the individual risk for progression or death and thus helping in treatment decision making.

## Data Availability

Datasets generated during the current study are not publicly available due to requirements of the ethical approval but are available from the corresponding author on reasonable request.
